# *N*,*N*′-(Phenyl­imino­dimethyl­ene)di­prop-2-enamide hemihydrate

**DOI:** 10.1107/S1600536809017784

**Published:** 2009-05-20

**Authors:** Dhanapal Tamilvendan, Ganesan Venkatesa Prabhu, Frank R. Fronczek, Nagarajan Vembu

**Affiliations:** a Department of Chemistry, National Institute of Technology, Tiruchirappalli 620 015, India; b Department of Chemistry, Louisiana State University, Baton Rouge, LA 70803-1804, USA; c Department of Chemistry, Urumu Dhanalakshmi College, Tiruchirappalli 620 019, India

## Abstract

In the title compound, C_14_H_17_N_3_O_2_·0.5H_2_O, the asymmetric unit consists of an *N*,*N*′-(phenyl­imino­dimethyl­ene)diprop-2-enamide mol­ecule and one half-mol­ecule of water, with the O atom of the latter having 2 site symmetry. The supra­molecular architecture is framed by the inter­play of two-dimensional networks of both O—H⋯O and N—H⋯O inter­actions supported by C—H⋯O and edge-to-face C—H⋯π inter­actions.

## Related literature

For a detailed description of Mannich bases and their applications, see: Friedrich *et al.* (1991 ▶); Bohme & Mannich (1955 ▶); Afsah *et al.* (2008 ▶); Terzioglu *et al.* (2006 ▶); Ravichandran *et al.* (2007 ▶); Pandeya *et al.* (2000 ▶). For hydrogen bonds, see: Desiraju & Steiner (1999 ▶); Jeffrey (1997 ▶). For hydrogen-bond motifs, see: Bernstein *et al.* (1995 ▶); Etter (1990 ▶).
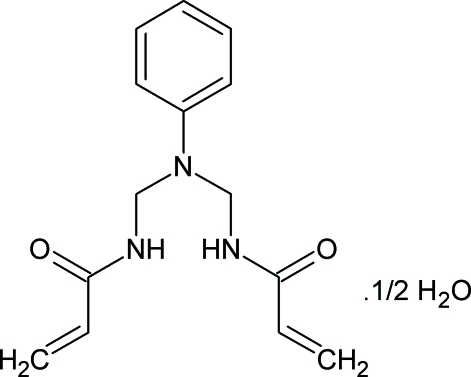


## Experimental

### 

#### Crystal data

C_14_H_17_N_3_O_2_·0.5H_2_O
*M* *_r_* = 268.31
Orthorhombic, 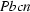

*a* = 17.074 (2) Å
*b* = 9.8366 (15) Å
*c* = 16.316 (2) Å
*V* = 2740.3 (6) Å^3^
*Z* = 8
Mo *K*α radiation
μ = 0.09 mm^−1^
*T* = 90 K
0.30 × 0.23 × 0.12 mm

#### Data collection

Nonius KappaCCD diffractometer with an Oxford Cryosystems Cryostream cooler
Absorption correction: none
9467 measured reflections
5065 independent reflections
3885 reflections with *I* > 2σ(*I*)
*R*_int_ = 0.030

#### Refinement

*R*[*F* ^2^ > 2σ(*F* ^2^)] = 0.045
*wR*(*F* ^2^) = 0.120
*S* = 1.02
5065 reflections
249 parameters
All H-atom parameters refined
Δρ_max_ = 0.39 e Å^−3^
Δρ_min_ = −0.28 e Å^−3^

### 

Data collection: *COLLECT* (Nonius, 2000 ▶); cell refinement: *DENZO* and *SCALEPACK* (Otwinowski & Minor, 1997 ▶); data reduction: *DENZO* and *SCALEPACK*; program(s) used to solve structure: *SHELXS97* (Sheldrick, 2008 ▶); program(s) used to refine structure: *SHELXL97* (Sheldrick, 2008 ▶); molecular graphics: *PLATON* (Spek, 2009 ▶); software used to prepare material for publication: *SHELXL97*.

## Supplementary Material

Crystal structure: contains datablocks I, global. DOI: 10.1107/S1600536809017784/lh2820sup1.cif

Structure factors: contains datablocks I. DOI: 10.1107/S1600536809017784/lh2820Isup2.hkl

Additional supplementary materials:  crystallographic information; 3D view; checkCIF report

## Figures and Tables

**Table 1:** Hydrogen-bond geometry (Å, °)

*D*—H⋯*A*	*D*—H	H⋯*A*	*D*⋯*A*	*D*—H⋯*A*
N5—H5⋯O11	0.876 (15)	2.318 (15)	3.0476 (11)	140.8 (12)
C4—H4*A*⋯O7	0.984 (13)	2.366 (13)	2.8089 (12)	106.5 (9)
C15—H15⋯O7	0.953 (14)	2.563 (14)	3.4922 (14)	165.1 (10)
O*W*—H*W*⋯O7^i^	0.845 (17)	1.990 (17)	2.8193 (9)	166.7 (16)
N1—H1⋯O11^ii^	0.891 (15)	2.089 (15)	2.9651 (11)	167.4 (14)
C2—H2*B*⋯*Cg*1^iii^	0.966 (13)	3.178	3.874	130.40
C8—H8⋯*Cg*1^iv^	0.966 (16)	2.571 (15)	3.4444 (12)	150.6 (13)

Symmetry codes: (i) 
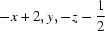
; (ii) 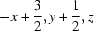; (iii) 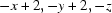; (iv) 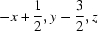. *Cg*1 is the centroid of the C14–C19 ring.

**Fig. 1.**
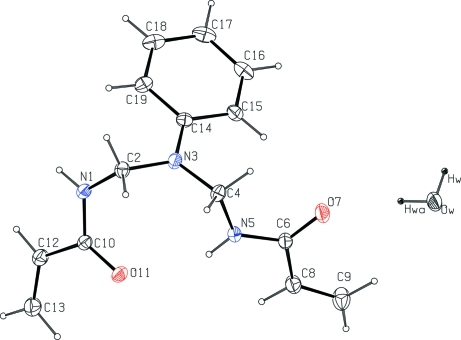
The asymmetric unit of (I) with the atoms labelled and displacement ellipsoids depicted at the 50% probability level for all non-H atoms. H-atoms are drawn as spheres of arbitrary radius.

**Fig. 2.**
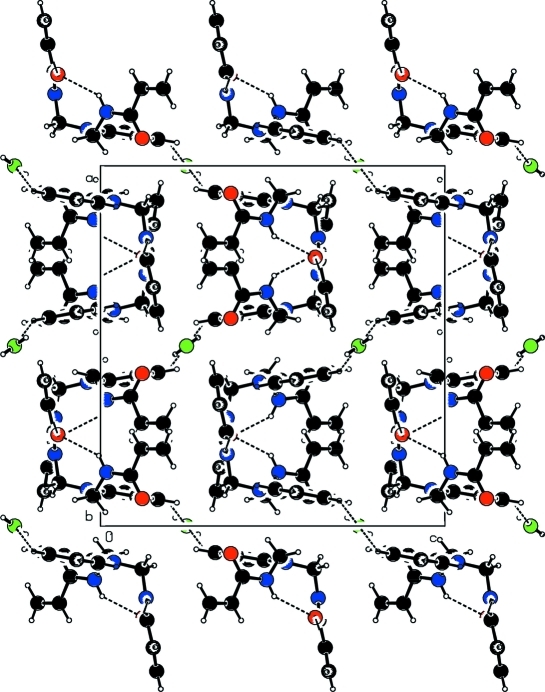
The molecular packing viewed along the b-axis. Dashed lines represent the O—H···O, N—H···O and C—H···O interactions within the lattice.

## supplementary crystallographic information

### Comment

The Mannich reaction is a three-component condensation in which a compound containing an active H atom (substrate) is allowed to react with an aldehyde or ketone and a primary or secondary amine with concomitant release of water to produce a new base known as a Mannich base, in which the active hydrogen is replaced by an aminomethyl group. The formation of both carbon-carbon and carbon-nitrogen bond in this aminomethylation process makes the Mannich reaction an extremely useful synthetic transformation. Mannich bases have wide application in the areas of pharmaceutics (Friedrich *et al.*, 1991) and macromolecular chemistry (Bohme & Mannich, 1955; Afsah *et al.*, 2008). Some Mannich bases have antimalarial, antiviral (Terzioglu *et al.*, 2006) properties while some other act as antihistamines, anti-inflammatories (Ravichandran *et al.*, 2007) and antimicrobials (Pandeya *et al.*, 2000). The present investigation is aimed at the elucidation of the molecular and crystal structure of the title compound which was obtained by the Mannich condendation of aniline, formaldehyde and acrylamide.

The asymmetric unit of (I) consists of *N,N*-[(phenylimino)dimethanediyl]bisprop-2-enamide and half a water molecule (Fig. 1).

The crystal structure of (I) is stabilized by O—H···O, N—H···O and C—H···O interactions. The range of H···O distances (Table 1) found in (I) agrees with those found for O—H···O & N—H···O (Jeffrey, 1997) and C—H···O hydrogen bonds (Desiraju & Steiner, 1999). Each of N5—H5···O11 and C15—H15···O7 interactions generate a ring motif of graph set (Bernstein *et al.*, 1995; Etter, 1990), S(8). An S(5) motif is formed by C4—H4A···O7. The Ow—Hw···O7^i^, N1—H1···O11^ii^ and N5—H5···O11 interactions together generate an extended two dimensional network along the base vectors, [0 1 0] & [1 0 0] and through the plane (0 0 - 1). The supramolecular architecture is completed by the interplay of two edge to face C—H···π interactions (Table 1).

### Experimental

7.1 g (0.1 mol) acrylamide and 10 ml (0.1*M*) of formaldehyde were dissolved in minimum amount of distilled water and the contents were mixed well to get a clear solution. 10 ml (0.1*M*) of aniline was added to the mixture in small installments with stirring. After 48 hr colorless solid was obtained which was washed with ethanol and dried at 343 K. The resulting organic compound was recrystallized from hot ethanol to yield the diffraction quality crystals of the title compound.

### Refinement

All H-atoms were located in difference maps and their positions and isotropic displacement parameters were freely refined.

### Crystal data

**Table:**

C_14_H_17_N_3_O_2_·0.5H_2_O	*D*_x_ = 1.301 Mg m^−^^3^
*M**_r_* = 268.31	Melting point: 398 K
Orthorhombic, *P**b**c**n*	Mo *K*α radiation, λ = 0.71073 Å
Hall symbol: -P 2n 2ab	Cell parameters from 34434 reflections
*a* = 17.074 (2) Å	θ = 2.5–33.0°
*b* = 9.8366 (15) Å	µ = 0.09 mm^−^^1^
*c* = 16.316 (2) Å	*T* = 90 K
*V* = 2740.3 (6) Å^3^	Fragment, colorless
*Z* = 8	0.30 × 0.23 × 0.12 mm
*F*(000) = 1144	

### Data collection

**Table:**

Nonius KappaCCD diffractometer with an Oxford Cryosystems Cryostream cooler	3885 reflections with *I* > 2σ(*I*)
Radiation source: fine-focus sealed tube	*R*_int_ = 0.030
graphite	θ_max_ = 33.0°, θ_min_ = 2.7°
ω and φ scans	*h* = −26→26
9467 measured reflections	*k* = −15→15
5065 independent reflections	*l* = −24→24

### Refinement

**Table:**

Refinement on *F*^2^	Primary atom site location: structure-invariant direct methods
Least-squares matrix: full	Secondary atom site location: difference Fourier map
*R*[*F*^2^ > 2σ(*F*^2^)] = 0.045	Hydrogen site location: difference Fourier map
*wR*(*F*^2^) = 0.120	All H-atom parameters refined
*S* = 1.02	*w* = 1/[σ^2^(*F*_o_^2^) + (0.0597*P*)^2^ + 0.7128*P*] where *P* = (*F*_o_^2^ + 2*F*_c_^2^)/3
5065 reflections	(Δ/σ)_max_ = 0.001
249 parameters	Δρ_max_ = 0.39 e Å^−^^3^
0 restraints	Δρ_min_ = −0.28 e Å^−^^3^

### Special details

**Table:**

Geometry. All e.s.d.'s (except the e.s.d. in the dihedral angle between two l.s. planes) are estimated using the full covariance matrix. The cell e.s.d.'s are taken into account individually in the estimation of e.s.d.'s in distances, angles and torsion angles; correlations between e.s.d.'s in cell parameters are only used when they are defined by crystal symmetry. An approximate (isotropic) treatment of cell e.s.d.'s is used for estimating e.s.d.'s involving l.s. planes.
Refinement. Refinement of *F*^2^ against ALL reflections. The weighted *R*-factor *wR* and goodness of fit *S* are based on *F*^2^, conventional *R*-factors *R* are based on *F*, with *F* set to zero for negative *F*^2^. The threshold expression of *F*^2^ > σ(*F*^2^) is used only for calculating *R*-factors(gt) *etc*. and is not relevant to the choice of reflections for refinement. *R*-factors based on *F*^2^ are statistically about twice as large as those based on *F*, and *R*- factors based on ALL data will be even larger.

### Fractional atomic coordinates and isotropic or equivalent isotropic displacement parameters (Å^2^)

**Table:**

	*x*	*y*	*z*	*U*_iso_*/*U*_eq_	
N1	0.80007 (5)	0.89440 (8)	0.13232 (5)	0.01594 (16)	
C2	0.88056 (6)	0.84769 (10)	0.12071 (6)	0.01569 (17)	
N3	0.89921 (5)	0.80690 (8)	0.03825 (5)	0.01545 (16)	
C4	0.91579 (6)	0.66584 (10)	0.02225 (6)	0.01665 (18)	
N5	0.85082 (5)	0.59623 (8)	−0.01804 (5)	0.01671 (16)	
C6	0.85921 (6)	0.52348 (9)	−0.08750 (6)	0.01635 (18)	
O7	0.92364 (4)	0.50903 (8)	−0.12139 (5)	0.02232 (17)	
C8	0.78566 (6)	0.46251 (11)	−0.11941 (6)	0.0210 (2)	
C9	0.78569 (7)	0.37886 (13)	−0.18250 (7)	0.0267 (2)	
C10	0.73876 (5)	0.80842 (9)	0.13486 (5)	0.01421 (17)	
O11	0.74626 (4)	0.68455 (7)	0.12367 (4)	0.01761 (15)	
C12	0.66234 (6)	0.87457 (10)	0.15303 (6)	0.01837 (19)	
C13	0.59676 (6)	0.80534 (12)	0.16321 (7)	0.0247 (2)	
C14	0.90493 (5)	0.90267 (10)	−0.02424 (6)	0.01502 (17)	
C15	0.91823 (5)	0.86297 (11)	−0.10565 (6)	0.01785 (19)	
C16	0.92620 (6)	0.96063 (12)	−0.16649 (6)	0.0226 (2)	
C17	0.92139 (6)	1.09817 (12)	−0.14964 (7)	0.0249 (2)	
C18	0.90668 (6)	1.13762 (11)	−0.06939 (7)	0.0229 (2)	
C19	0.89752 (6)	1.04206 (10)	−0.00757 (7)	0.01868 (19)	
OW	1.0000	0.37093 (12)	−0.2500	0.0356 (3)	
H1	0.7899 (9)	0.9829 (15)	0.1371 (9)	0.028 (4)*	
H2A	0.8901 (7)	0.7705 (14)	0.1559 (8)	0.018 (3)*	
H2B	0.9140 (7)	0.9206 (14)	0.1393 (8)	0.015 (3)*	
H4A	0.9616 (8)	0.6566 (13)	−0.0140 (8)	0.018 (3)*	
H4B	0.9274 (7)	0.6225 (13)	0.0743 (8)	0.017 (3)*	
H5	0.8046 (9)	0.6058 (14)	0.0045 (9)	0.027 (3)*	
H8	0.7378 (9)	0.4864 (15)	−0.0914 (10)	0.035 (4)*	
H9A	0.7377 (8)	0.3357 (15)	−0.2005 (9)	0.029 (4)*	
H9B	0.8348 (9)	0.3542 (15)	−0.2119 (10)	0.036 (4)*	
H12	0.6636 (9)	0.9714 (16)	0.1589 (10)	0.034 (4)*	
H13A	0.5471 (8)	0.8504 (15)	0.1751 (8)	0.027 (3)*	
H13B	0.5952 (10)	0.7040 (19)	0.1577 (11)	0.045 (5)*	
H15	0.9212 (7)	0.7696 (15)	−0.1208 (8)	0.018 (3)*	
H16	0.9353 (9)	0.9338 (15)	−0.2219 (9)	0.031 (4)*	
H17	0.9294 (9)	1.1688 (16)	−0.1936 (10)	0.038 (4)*	
H18	0.9027 (8)	1.2309 (16)	−0.0557 (9)	0.029 (4)*	
H19	0.8871 (8)	1.0736 (14)	0.0458 (9)	0.022 (3)*	
HW	1.0232 (10)	0.4232 (17)	−0.2832 (11)	0.048 (5)*	

### Atomic displacement parameters (Å^2^)

**Table:**

	*U*^11^	*U*^22^	*U*^33^	*U*^12^	*U*^13^	*U*^23^
N1	0.0168 (4)	0.0121 (3)	0.0189 (4)	−0.0001 (3)	0.0009 (3)	−0.0017 (3)
C2	0.0163 (4)	0.0169 (4)	0.0138 (4)	0.0001 (3)	−0.0014 (3)	−0.0017 (3)
N3	0.0195 (4)	0.0131 (3)	0.0137 (3)	0.0013 (3)	0.0007 (3)	−0.0011 (3)
C4	0.0184 (4)	0.0141 (4)	0.0175 (4)	0.0024 (3)	−0.0011 (3)	−0.0010 (3)
N5	0.0166 (4)	0.0167 (4)	0.0168 (4)	−0.0006 (3)	0.0038 (3)	−0.0035 (3)
C6	0.0181 (4)	0.0153 (4)	0.0157 (4)	0.0014 (3)	0.0026 (3)	−0.0010 (3)
O7	0.0176 (3)	0.0259 (4)	0.0235 (4)	0.0004 (3)	0.0054 (3)	−0.0080 (3)
C8	0.0177 (4)	0.0243 (5)	0.0210 (5)	−0.0002 (4)	0.0027 (3)	−0.0048 (4)
C9	0.0219 (5)	0.0343 (6)	0.0241 (5)	0.0014 (4)	−0.0020 (4)	−0.0094 (4)
C10	0.0172 (4)	0.0133 (4)	0.0122 (4)	−0.0002 (3)	−0.0010 (3)	0.0001 (3)
O11	0.0213 (3)	0.0118 (3)	0.0197 (3)	0.0002 (2)	0.0004 (3)	−0.0009 (2)
C12	0.0191 (4)	0.0148 (4)	0.0212 (5)	0.0011 (3)	0.0008 (3)	−0.0012 (3)
C13	0.0204 (5)	0.0241 (5)	0.0294 (5)	−0.0018 (4)	0.0037 (4)	−0.0072 (4)
C14	0.0119 (4)	0.0163 (4)	0.0169 (4)	−0.0010 (3)	−0.0011 (3)	0.0011 (3)
C15	0.0154 (4)	0.0199 (5)	0.0182 (4)	0.0000 (3)	0.0031 (3)	0.0010 (4)
C16	0.0184 (4)	0.0305 (6)	0.0189 (5)	−0.0014 (4)	0.0038 (4)	0.0055 (4)
C17	0.0208 (5)	0.0264 (5)	0.0274 (5)	−0.0039 (4)	0.0000 (4)	0.0116 (4)
C18	0.0206 (5)	0.0177 (5)	0.0305 (6)	−0.0034 (3)	−0.0044 (4)	0.0054 (4)
C19	0.0180 (4)	0.0164 (4)	0.0216 (5)	−0.0016 (3)	−0.0032 (3)	−0.0002 (3)
OW	0.0567 (8)	0.0170 (5)	0.0330 (7)	0.000	0.0289 (6)	0.000

### Geometric parameters (Å, °)

**Table:**

N1—C10	1.3465 (12)	C10—O11	1.2387 (11)
N1—C2	1.4613 (13)	C10—C12	1.4878 (14)
N1—H1	0.891 (15)	C12—C13	1.3210 (15)
C2—N3	1.4397 (12)	C12—H12	0.957 (16)
C2—H2A	0.966 (13)	C13—H13A	0.976 (14)
C2—H2B	0.966 (13)	C13—H13B	1.001 (19)
N3—C14	1.3915 (12)	C14—C15	1.4030 (14)
N3—C4	1.4400 (12)	C14—C19	1.4036 (14)
C4—N5	1.4599 (12)	C15—C16	1.3880 (14)
C4—H4A	0.984 (13)	C15—H15	0.953 (14)
C4—H4B	0.970 (13)	C16—C17	1.3830 (17)
N5—C6	1.3480 (12)	C16—H16	0.954 (15)
N5—H5	0.876 (15)	C17—C18	1.3886 (17)
C6—O7	1.2394 (12)	C17—H17	1.008 (16)
C6—C8	1.4859 (14)	C18—C19	1.3876 (15)
C8—C9	1.3178 (15)	C18—H18	0.947 (16)
C8—H8	0.966 (16)	C19—H19	0.942 (14)
C9—H9A	0.969 (14)	OW—HW	0.845 (17)
C9—H9B	0.996 (16)		
			
C10—N1—C2	122.52 (8)	H9A—C9—H9B	117.4 (12)
C10—N1—H1	117.3 (10)	O11—C10—N1	122.20 (9)
C2—N1—H1	120.2 (10)	O11—C10—C12	123.38 (9)
N3—C2—N1	114.64 (8)	N1—C10—C12	114.41 (8)
N3—C2—H2A	107.4 (8)	C13—C12—C10	122.88 (9)
N1—C2—H2A	109.2 (8)	C13—C12—H12	121.3 (9)
N3—C2—H2B	111.7 (8)	C10—C12—H12	115.8 (9)
N1—C2—H2B	106.4 (8)	C12—C13—H13A	121.8 (9)
H2A—C2—H2B	107.3 (11)	C12—C13—H13B	121.6 (10)
C14—N3—C2	120.77 (8)	H13A—C13—H13B	116.6 (13)
C14—N3—C4	120.38 (8)	N3—C14—C15	121.10 (9)
C2—N3—C4	118.78 (8)	N3—C14—C19	120.87 (9)
N3—C4—N5	112.59 (8)	C15—C14—C19	118.03 (9)
N3—C4—H4A	110.7 (7)	C16—C15—C14	120.02 (10)
N5—C4—H4A	106.8 (7)	C16—C15—H15	118.5 (8)
N3—C4—H4B	107.8 (8)	C14—C15—H15	121.5 (8)
N5—C4—H4B	110.0 (8)	C17—C16—C15	121.95 (10)
H4A—C4—H4B	108.8 (11)	C17—C16—H16	118.0 (9)
C6—N5—C4	123.15 (8)	C15—C16—H16	120.1 (9)
C6—N5—H5	120.4 (10)	C16—C17—C18	118.13 (10)
C4—N5—H5	116.4 (9)	C16—C17—H17	121.7 (9)
O7—C6—N5	122.03 (9)	C18—C17—H17	120.2 (9)
O7—C6—C8	123.19 (9)	C19—C18—C17	121.10 (10)
N5—C6—C8	114.78 (8)	C19—C18—H18	118.4 (9)
C9—C8—C6	121.70 (9)	C17—C18—H18	120.5 (9)
C9—C8—H8	121.4 (9)	C18—C19—C14	120.72 (10)
C6—C8—H8	116.9 (9)	C18—C19—H19	118.1 (8)
C8—C9—H9A	120.7 (9)	C14—C19—H19	121.2 (8)
C8—C9—H9B	121.9 (9)		
			
C10—N1—C2—N3	75.00 (12)	N1—C10—C12—C13	−175.46 (10)
N1—C2—N3—C14	70.15 (11)	C2—N3—C14—C15	−175.96 (8)
N1—C2—N3—C4	−112.95 (9)	C4—N3—C14—C15	7.19 (13)
C14—N3—C4—N5	−78.90 (10)	C2—N3—C14—C19	4.02 (13)
C2—N3—C4—N5	104.19 (10)	C4—N3—C14—C19	−172.83 (8)
N3—C4—N5—C6	127.89 (10)	N3—C14—C15—C16	−177.94 (9)
C4—N5—C6—O7	1.15 (15)	C19—C14—C15—C16	2.09 (13)
C4—N5—C6—C8	−179.03 (9)	C14—C15—C16—C17	−0.07 (15)
O7—C6—C8—C9	5.99 (17)	C15—C16—C17—C18	−1.19 (16)
N5—C6—C8—C9	−173.83 (11)	C16—C17—C18—C19	0.39 (15)
C2—N1—C10—O11	−3.45 (14)	C17—C18—C19—C14	1.67 (15)
C2—N1—C10—C12	175.89 (8)	N3—C14—C19—C18	177.14 (9)
O11—C10—C12—C13	3.88 (16)	C15—C14—C19—C18	−2.88 (14)

### Hydrogen-bond geometry (Å, °)

**Table:**

*D*—H···*A*	*D*—H	H···*A*	*D*···*A*	*D*—H···*A*
N5—H5···O11	0.876 (15)	2.318 (15)	3.0476 (11)	140.8 (12)
C4—H4A···O7	0.984 (13)	2.366 (13)	2.8089 (12)	106.5 (9)
C15—H15···O7	0.953 (14)	2.563 (14)	3.4922 (14)	165.1 (10)
OW—HW···O7^i^	0.845 (17)	1.990 (17)	2.8193 (9)	166.7 (16)
N1—H1···O11^ii^	0.891 (15)	2.089 (15)	2.9651 (11)	167.4 (14)
C2—H2B···Cg1^iii^	0.966 (13)	3.178	3.874	130.40
C8—H8···Cg1^iv^	0.966 (16)	2.571	3.444	150.57

Symmetry codes: (i) −*x*+2, *y*, −*z*−1/2; (ii) −*x*+3/2, *y*+1/2, *z*; (iii) −*x*+2, −*y*+2, −*z*; (iv) −*x*+1/2, *y*−3/2, *z*.
